# The effectiveness of hydrogel matrix containing nitric oxide, silver nanoparticles, vancomycin, and ciprofloxacin on methicillin-resistant *Staphylococcus aureus* and *Pseudomonas aeruginosa* biofilm isolated from patients with chronic rhinosinusitis

**DOI:** 10.1186/s40001-025-03282-z

**Published:** 2025-10-29

**Authors:** Zahra Chegini, Aref Shariati, Shahin Rajaeih, Mohammad Yousef Alikhani, Maliheh Safaiee, Mohammadreza Arabestani, Mehdi Azizi

**Affiliations:** 1 Department of Microbiology, School of Medicine, Hamadan University of Medical Sciences, Hamadan, Iran; 2 Infectious Diseases Research Center (IDRC), Arak University of Medical Sciences, Arak, Iran; 3 ENT and Head and Neck Research Center and Department, The Five Senses Health Institute, Iran University of Medical Sciences, Tehran, Iran; 4 Department of Organic Chemistry, Faculty of Chemistry and Petroleum Sciences, Bu-Ali Sina University, Hamedan, Iran; 5 Infectious Diseases Research Center, Avicenna Institute of Clinical Sciences,, Hamadan University of Medical Sciences, Hamadan, Iran; 6 Department of Tissue Engineering and Biomaterials, School of Advanced Medical Sciences and Technologies, Hamadan University of Medical Sciences, Hamadan, Iran

**Keywords:** Antimicrobial resistance, Silver nanoparticles, Chronic rhinosinusitis, Biofilms, Methicillin-resistant *Staphylococcus aureus*, *Pseudomonas aeruginosa*

## Abstract

**Background:**

Many scientists are studying chronic rhinosinusitis (CRS) due to its high relapse incidence and drug resistance. We investigated the antibiofilm activity and sustained co-release of nitric oxide, silver nanoparticles, vancomycin, and ciprofloxacin in thermosensitive hydrogel (HyNSVC) for bacterial CRS.

**Methods:**

To identify and isolate *Pseudomonas aeruginosa* and methicillin-resistant *Staphylococcus aureus* (MRSA), samples were taken from 10 patients with CRS who underwent Functional Endoscopic Sinus Surgery (FESS), and then they were diagnosed using culture and molecular methods. The HyNSVC was synthesized, and its physicochemical characteristics were evaluated using different methods. The broth microdilution assay and the MTP (microtiter plate) method assessed the minimum inhibitory concentration (MIC) and antibiofilm effects. The inhibitory impact of HyNSVC on the expression of biofilm-associated genes was evaluated using real-time PCR. The cytotoxic effect of HyNSVC on the human epithelial cell line A549 was assessed.

**Results:**

The results of the SEM showed that the hydrogels have a porous structure and that the pores are interconnected. The molecular weights and polydispersity index (PDI) of the copolymer determined using gel permeation chromatography (GPC) were 5759 Daltons, 6270 Daltons, and 1.089, respectively. The anticipated molecular weight determined by H-NMR spectroscopy was around 5947 Daltons. Also, the porosity of the hydrogels was in the range of 80–90%. The HyNSVC MIC was 250 µg/ml for both bacteria. The 2 × MIC of HyNSVC reduced mature biofilm by 62% in *P. aeruginosa* and 68.1% in MRSA. The presence of HyNSVC did not significantly reduce gene expression. Finally, the MTT experiment showed no toxicity against A549 cells at the MIC concentration.

**Conclusion:**

Our findings illustrated the efficacy of employing HyNSVC for drug release regulation and antibiofilm activity for bacterial infection, suggesting a promising treatment for CRS.

**Supplementary Information:**

The online version contains supplementary material available at 10.1186/s40001-025-03282-z.

## Introduction

Chronic rhinosinusitis (CRS) is characterized by sinus inflammation symptoms that persist for at least two months. The pathogenesis of CRS is considered complex, encompassing fungal colonization, bacterial infection, environmental or aspirin sensitivity, and idiopathic inflammation [1]. The aerobic and anaerobic microbiota inhabit healthy sinuses. Numerous opportunistic pathogens exist in low quantities within healthy sinuses and hence possess the capacity to induce illnesses following an abrupt disruption of the stable baseline microbial ecology. According to this idea, CRS may arise from a specific sequence of interdependent occurrences: compromised mucus clearance and disturbance of the stable microbiota [2, 3]. *Pseudomonas aeruginosa* and *Staphylococcus aureus*, particularly methicillin-resistant *S. aureus* (MRSA), are the predominant microorganisms associated with CRS patients, contributing to the duration and severity of CRS and the failure of antibiotic treatment through biofilm formation [2]. Biofilms are complicated and well-organized communities of microbes that produce a matrix composed of exopolysaccharides [4]. The dense configuration of extracellular polymeric substances (EPS) produced by bacteria can obstruct the penetration of host immune cells and antibiotics, allowing bacteria to persist for prolonged durations [5]. Currently, antibiotics are regarded as a standard initial treatment for bacterial rhinosinusitis. However, biofilms are recognized as a prevalent and significant contributor to chronic illnesses; they necessitate antibiotic doses up to 1,000 times greater for effective treatment compared to planktonic cells, hence complicating eradication efforts [6]. To this end, different therapies are being formulated to mitigate biofilm-related issues in CRS patients, including novel antibiotics, their combinations and adjuvants, bacteriophage therapy, and nanostructure functionalization [7, 8]. Moreover, existing systemic treatments, such as oral or intravenous antimicrobials and corticosteroids, exhibited considerable side effects and often proved ineffective in numerous people. This issue has prompted researchers to investigate the localized administration of topical treatments.

Localized medication administration may markedly diminish systemic adverse effects and enhance the effectiveness of CRS treatments [9]. Nanotechnology-based drug delivery can surmount specific anatomical, physiological, pharmacological, and therapeutic obstacles linked to conventional treatment. The potential benefits of nanoparticles encompass enhanced medication solubility and stability, augmented bioavailability at the targeted site, and extended duration of action through regulated release rates. The mentioned advantages may yield negligible side effects and a more convenient administration method, resulting in greater patient adherence and enhanced treatment outcomes [10]. Metal nanomaterials, including silver nanoparticles (AgNPs), can release metal ions that inactivate bacteria [11]. When AgNPs come into contact with bacteria, they bind to their cell walls. This interaction produces concentrated silver ions (Ag^+^) at the contact between the nanoparticles and the bacterium. Consequently, Ag^+^ can cause oxidative stress, disruption of cell membrane integrity, loss of metabolic function, inhibition of respiratory chain enzymes, and DNA damage in bacterial cells [12, 13].

Furthermore, nitric oxide (NO) is a free radical molecule produced from the amino acid L-arginine by three specific isoforms of NO synthase. Healthy individuals exhibit elevated NO levels in the sinuses, contributing to antibacterial and antiviral activities and maintaining a relatively sterile environment while augmenting the mucociliary clearance function [14]. Patients with CRS exhibited reduced sinonasal NO levels [15]. Recent investigations have shown elevated NO concentrations inhibit *S. aureus* biofilm proliferation [16]. Hence, combining antibiotics and other antibacterial compounds such as NO can improve AgNPs function against CRS infection.

However, the critical point is that in treating CRS, we need an appropriate drug delivery device that can lead to longer retention of compounds and drugs in the sinus area due to mucociliary activity in the nasal area. For this purpose, hydrogels possess significant potential in targeted drug delivery systems and protein–ligand recognition [17, 18]. The administration of medications in situ, facilitated by injectable thermosensitive hydrogel, is a compelling method for localized drug delivery [19]. Poly(ethylene glycol)–poly(propylene glycol)–poly(ethylene glycol) (PEG–PPG–PEG) copolymers are commercially available thermosensitive hydrogels that have been extensively utilized in experimental medicine. poly(e-caprolactone) (PCL) and poly(ethylene glycol) (PEG) are biocompatible polymers utilized in many FDA-approved items [20].

The current study aimed to construct and assess a hydrogel matrix incorporating NO, AgNPs, vancomycin, and ciprofloxacin for sustained drug release via hydrogel, targeting the management of *P. aeruginosa* and *S. aureus* biofilms isolated from CRS patients. In this study for the first time, antibacterial, anti-biofilm, and anti-inflammatory agents have been incorporated into a hydrogel for use against the most important bacteria in CRS patients.

## Materials and methods

### Research materials

Sigma-Aldrich Canada (Oakville, ON) supplied sodium nitrite (NaNO2), reduced glutathione (GSH), N-(1-naphthyl) ethylenediamine dihydrochloride, sulfanilamide, and other analytical solvents. PC2 and PC5 phytochelatins were purchased from AnaSpec Inc. (San Jose, CA). Poly (vinyl methyl ether-co-maleic anhydride) (PVMMA, Gantrez AN-169, MW: 2,000,000) and poly (vinyl pyrrolidone) (PVP Plasdone K-90, MW: 1,300,000) were gratefully supplied by ISP (Wayne, NJ).

### Copolymer synthesis and purification

The 20.0 g (0.175 mol) of caprolactone, 10.0 g (0.01 mol) of PEG, and 0.15 g of Sn(Oct)_2_ were added in a round bottom container with three holes and then degassed for half an hour. Under dry nitrogen atmosphere, the mixture was placed at 130 °C for 6 h. Next, the copolymer was cooled to room temperature. Dichloromethane and ether were used to purify the obtained PCL–PEG–PCL copolymer. Next, to connect dopamine dihydrochloride (to increase the surface adhesion property of the final product), the PCL–PEG–PCL copolymer was first treated with sodium hydroxide solution for 24 h to create hydroxyl functional groups on the surface of the copolymer. After that, the polydopamine coating was done by dropwise deposition of Tris buffer solution containing dopamine at pH 8.5 on the PCL–PEG–PCL copolymer treated with NaOH. At this stage, FTIR was used to confirm the functional groups and successful surface modification[17].

#### Synthesis of S-nitrosoglutathione (GSNO)

The compound GSNO was prepared by reacting GSH with sodium nitrite in an acidic environment protected from light. For this purpose, in a double-necked round-bottomed flask placed in a stirring ice bath, a specified amount of GSH (154 mg, 0.5 mmol) in 5 ml of 0.1 normal HCl, a specified amount of NaNO_2_ (35 mg, 0.5 mmol) was added. The reaction for GSNO production was conducted with a high efficiency of more than 80%. The resultant GSNO stock solution was shielded from light using aluminum foil and utilized immediately without additional purification [21].

#### Conjugation of GSNO to PVMMA

The connection of GSNO to PVMMA occurred through a heterogeneous reaction of GSNO with PVMMA. For this purpose, 500 mg of PVMMA was first homogeneously dissolved in 10 ml of acetone. The synthetic GSNO solution of the above step (2 mL) was added dropwise to the PVMMA solution under stirring in an ice bath. After that, the mixture was poured into a Teflon container and placed under air drying at room temperature and in the dark to collect and dry the final product in the form of GSNO-PVMMA as a pink powder with a loading of 11.86% by weight of GSNO [21].

### Fabrication of the hydrogel nanocomposite

To create PCL–PEG–PCL hydrogel at a concentration of 20 wt%, PCL–PEG–PCL was dissolved in deionized water at 25 °C (room temperature). In the case of AgNPs and drugs, GSNO-PVMMA, AgNPs, and drugs were mixed to form a homogeneous solution before the complex formation. The AgNPs used in this section were synthesized via a green synthesis method using phycocyanin in our previous study [5]. The PCL–PEG–PCL solution was then rapidly added to the GSNO–PVMMA solution while vigorously stirring. The resulting mixture’s viscosity grew dramatically when the complex was formed through intermolecular hydrogen bonding, resulting in a pink gel-like product, the degree of which varied depending on the composition. The artificially created hydrogels were frozen at − 80 °C for 6 h. Subsequently, they were subjected to lyophilization using a freeze dryer (Telstar, Terrassa, Spain) at − 54 °C for 24 h. This process was carried out to develop scaffolds with a porous structure.

### Characterization of synthesized polymer

The powdered HyNSVC was evaluated via FTIR spectroscopy (Tensor 27, Bruker Co) within a frequency range of 4000–400 cm–1 and a resolution of 4 cm–1, and the analysis was done using the KBr pellet method. GPC (Agilent 110 HPLC, Santa Clare, CA) was employed to ascertain the copolymer’s molecular weight and polydispersity. Tetrahydrofuran (THF) dissolved the sample at 1–2 mg/ml. Two Waters Styragel HT columns and a linear column were used to elute THF at a 1.0 ml/min rate. The temperature of the column and the outside was maintained at 35C. Standard polystyrene samples with a restricted molecular weight distribution were used to calculate the molecular weights of the samples. The Varian 400 spectrometer (Varian, Palo, CA) was used to record H-NMR spectra (in CDCl3) at 400 MHz to determine the copolymer’s chemical composition and macromolecular weight [21].

### Characterization of the hydrogel

The samples underwent freeze-drying, after which the resulting dried samples were subsequently sliced to a diameter of 7 mm. The hydrogels’ ultrastructure was examined using a FESEM–EDX (field emission scanning electron microscopy with energy dispersive *X*-ray spectroscopy, AIS2100, Seron Technology) apparatus operating at an accelerating voltage of 20 kV. Before imaging, the hydrogels were coated with a layer of gold using a sputter coater for 250 s. The powdered HyNSVC was evaluated via FTIR spectroscopy (Tensor 27, Bruker Co) within a frequency range of 4000–400 cm–1 and a resolution of 4 cm–1, and the analysis was done using the KBr pellet method [22].

The swelling ratios were determined using a gravimetric technique. The dried hydrogels were submerged in a phosphate-buffered saline (PBS) solution with a pH of 7.4. Following a predetermined duration, tissue paper was employed to meticulously eliminate any excess water that had formed on the surface of the hydrogel. The hydrogel masses were measured using an analytical balance. The swelling ratio was determined by using the subsequent mathematical expression:

$$\text{Swelling }\left(\text{\%}\right)=(\frac{\text{Ms}-\text{Md}}{\text{Md}})\times 100.$$

The mass of the swollen (MS) and dried (Md) samples at various intervals is denoted by the MS and Md variables, respectively.

### Swelling studies

The swelling studies were conducted at various temperatures in PBS solution. The equilibrium swelling ratio (ESR) was determined using the gravimetric method. A sample of a specific weight was submerged in solutions of varying temperatures until swelling equilibrium was attained; the surplus surface liquid was eliminated by blotting, and the swelled sample was subsequently weighed. The ESR was computed using the following equation:

$${\text{ESR }} = {\text{w}}_{{\text{w}}} /{\text{w}}_{{\text{d}}} ,$$

where w_w_ and wd represent the sample weights (g) in the swollen and dried phases, respectively. The sample was transferred between identical tubs maintained at either 37 °C or 25 °C for swelling and deswelling tests. Before weighing, the sample was periodically extracted from the media and blotted with filter paper to eliminate surplus surface moisture.

### Drug loading

The loading process involved immersing approximately 0.5 g of dry hydrogel in 100 mL of a drug solution with known concentrations (50 mg/ml) in an appropriate glass container; the temperature was maintained at 37 °C, and the solution was gently agitated. Initial research indicated that a 24 h loading period was adequate [23]. The equilibrium concentration of the medication in the media was quantified using a UV spectrophotometer (UV-260, Shimadzu). The drug loading capacity of the hydrogel was assessed by calculating the difference between the beginning and final quantities in the medium.

### Drug release

The drug-loaded hydrogel (about 0.5 g) was weighed in duplicate and introduced into 250 mL of PBS (pH 7.4) as the release medium. The drug release was evaluated under mechanical agitation at 60 rpm. Five milliliters of the discharged solution were collected at specified intervals and filtered through a 0.45-micron membrane filter. An equivalent volume of fresh medium was substituted. A spectrophotometer (UV-260, Shimadzu) assessed the quantity of medication released. The results were given as cumulative release over time:

$${\text{Cumulative released }}\left( \% \right) \, = {\text{ Mt}}/{\text{M}}\infty \, \times {1}00,$$

where Mt represents the quantity of pharmaceuticals released from the hydrogel at time t, and M∞ denotes the expected quantity of drugs encapsulated within the hydrogel.

### Cytotoxicity study (MTT assay)

In this study, the A549 cell line was cultivated at a density of 1 × 10^4^ cells in 150 μl of HyNSVC within DMEM/F12 media supplemented with 10% FBS and antibiotics for 24 h. The culture was preserved in a humidified incubator at 37 °C with a 5% CO_2_ concentration. Following 24, 48, and 72 h incubation, the culture media were removed from the 96-well plate. Subsequently, 0.2 ml of MTT solution (0.5 mg/ml) was added to each well. The plate was subsequently incubated at 37 °C for 4 h. After incubation, the MTT solution was eliminated, and 0.1 ml of DMSO was introduced to each well. The absorbance was measured at approximately 570 nm. The average values of the triplicate wells for each sample were provided [24, 25].

### Microbiological analysis of patient samples

Participants in this study were CRS patients who were sent to two tertiary academic hospitals in Tehran, Iran, between June and November 2023 and were eligible for functional endoscopic sinus surgery (FESS). Under general anesthesia, individuals underwent endoscopic endonasal operations. Under endoscopic guidance, sterilized rayon-tipped swabs coated with a flat sheet were used to take a sample inside the sphenoidal sinus surface mucosa to acquire a sample without contaminating the nasal cavity [26]. The coated swab was placed inside the sphenoid sinus ostium. After being forced uncovered into the sinus, the swab was pulled back into the cover and removed through the nose. The tip of each swab was aseptically removed and placed on ice in a sterile tube holding 1.5 mL of brain heart infusion (BHI) as soon as the material was collected. The swabs were promptly brought to the lab on ice and cultured on cetrimide and mannitol salt agar. For 24 h, agar plates were incubated at 37 °C to promote growth. Biochemical assays, including Gram-staining, oxidase, oxidation-fermentation (OF) test, pigment generation in Mueller–Hinton agar, the Kligler Iron Agar (KIA) tests, catalase response, and mannitol fermentation, were used to identify all positive cultures [27]. MRSA isolates were detected using a cefoxitin disc (30 μg) under Clinical and Laboratory Standards Institute (CLSI) guidelines [28]. Also, in this study, after DNA extraction, bacteria in the samples were confirmed through real-time PCR using specific primers and probes, as in our previous study (Supplementary File, Table S3) [26].

### Bacteria growth inhibition by broth microdilution assay

The minimum inhibitory concentration (MIC) of the HyNSVC was ascertained utilizing the methodology employed in our prior research [5]. Bacterial isolates were incubated overnight at 37 °C. The number of bacterial cells was set to 0.5 McFarland turbidity using a spectrophotometer at the optical density (OD) of 625 nm. Then, 50 μL of the provided bacterial suspensions was added to 950 μL of Mueller–Hinton broth (MHB). Following this, serial dilutions of HyNSVC, commencing at a concentration ranging from 1000 to 62.5 µg/mL, were added to 96-well polystyrene plates. Each well was inoculated with 10 μL of diluted bacteria in MHB, achieving a final 5 × 10^5^ CFU/mL concentration. The MIC was determined to be the minimal concentration of HyNSVC that inhibited the growth of the bacteria. The studies were performed in triplicate with *S. aureus* ATCC 25923 and *P. aeruginosa* ATCC 27853, with uninoculated MHB medium considered positive and negative controls, respectively [5]. Notably, the antibacterial activity of natural compounds was evaluated against two different MRSA strains and *P. aeruginosa*.

### Minimal biofilm inhibitory and eradication concentration (MBIC and MBEC)

Five microliters of each bacterial stock were inoculated into 5 mL of MH broth and incubated at 37 °C for 24 h to evaluate the biofilm production of bacterial isolates. Optical density was determined at 600 nm, and the MRSA and *P. aeruginosa* isolates were adjusted in TSB to attain a bacterial concentration of 10^6^ CFU/mL. Subsequently, 200 μL of TSB was introduced into sterile flat-bottom 96-well microplates. The microplate was thereafter incubated at 37 °C for 24 h. After this duration, the microplate was rinsed thrice with 200 μL of sterile distilled water and then inverted onto paper towels to dry for 5 min at ambient temperature. The biofilm was stabilized by adding 150 μL of methanol to each well for 20 min at room temperature. Subsequently, the microplate contents were removed, and the plate was allowed to dry in an inverted orientation on paper towels at room temperature. The wells were subsequently stained with 200 μL of 0.05% crystal violet for 5 min. The wells were thereafter rinsed thrice with 200 μL of distilled water. Subsequently, 200 μL of 95% ethanol was introduced to each well, and absorbance was quantified at 600 nm utilizing a microplate reader. The assays were conducted in triplicate. Based on the obtained optical density (OD) values, the isolates were categorized as non-biofilm producers (OD_test_ ≤ OD_control_), weak producers (OD_control_ < OD_test_ ≤ 2 × OD_control_), moderate producers (2 × OD_control_ < OD_test_ ≤ 4 × OD_control_), or strong biofilm producers (OD_test_ > 4 × OD_control_), with OD_test_ denoting the optical density of the MRSA and *P. aeruginosa* isolates and OD_control_ indicating the optical density of the control strain. The biofilm development test was replicated thrice [29].

A strain of *P. aeruginosa* and MRSA that exhibited strong biofilm formation and control bacteria were utilized for subsequent testing. To assess the MBIC effect of HyNSVC, 100 µl of chosen bacterial cultures (10^6^ CFU/ml) diluted with TSB were introduced into a sterile 96-well microtiter plate. Subsequently, 100 µl of the serial dilutions of different concentrations of HyNSVC (1/2 MIC) were introduced into each well, and the plate was incubated overnight at 37 °C for 24 h with shaking at 75 rpm. After the incubation period, the contents of each well were discarded. After washing the wells 3 times with 200 μl of sterile PBS, the established biofilms were preserved with 200 μL of absolute methanol for 5 min. In the next step, the contents of the wells were drained, and the wells were air-dried again. Then, the wells were stained with 0.05% crystal violet for 5 min, the solution was drained, and the wells were washed three times with PBS and air-dried again. Finally, 200 μL of absolute ethanol was added to each well, and the optical densities were measured using a microplate ELISA reader at 600 nm in the final phase:

$${\text{Biofilm inhibition \% }} = \, \left[ {{1} - \left( {{\text{OD test }}/{\text{ OD control}}} \right)} \right]\, \times \,{1}00.$$

The efficiency of synthesized HyNSVC in biofilm removal was assessed using MBEC. The analyzed isolates were initially permitted to develop a pre-formed biofilm for 24 h. The well contents were removed and rinsed three times with saline solution. Simultaneously, 100 μL of 2 × MIC and 4 × MIC of HyNSVC was applied to the wells at a volume of 100 μL and incubated at 37 °C for 24 h. After overnight incubation, the content of the wells was gently aspirated and washed 3 times with PBS. Finally, the MTP method measured the quantity of formed biofilm, as explained above. The positive control consisted of microorganisms in TSB-glucose devoid of HyNSVC. The biofilm clearance % was determined using the absorbance described in the equation above [5, 30].

### Evaluating the influence of HyNSVC on the expression of genes associated with biofilm formation

The effect of HyNSVC formulation on biofilm gene expression was tested using real-time PCR. All primers (Table 1) and RNA extraction methods were previously reported [26]. The isolates were first treated with sub-MIC of HyNSVC, and total RNAs were extracted and reverse transcribed into cDNA using random hexamer primers. Real-time PCR was performed using the SYBR green qPCR master mix to measure biofilm-associated gene expression (*icaR* and *lasR*). The cycle threshold (CT) values of the mentioned genes were compared to 16S rRNA, a housekeeping gene, to standardize biofilm gene expression. The relative expression of genes was evaluated using 2^−∆∆Ct^ [26].

**Table 1:** List of specific primers

Gene	Sequence
*Ica*-F	AAAGATGTAGGTTATTGGGATACTGACA
*Ica*-R	CATAGAGCACGTGGTTCGTACTTAA
*lasR*- F	AAGTGGAAAATTGGAGTGGAG
*lasR*-R	GTAGTTGCCGACGACGATGAAG
*algD*-F	ATGCGAATCAGCATCTTTGGT
*algD*-R	CTACCAGCAGATGCCCTCGGC
16 s rRNA-F	ACTTCGGGAAACCGGAGC
16 s rRNA-R	ACCGTGTCTCAGTTCCAG

### Statistical analysis

All statistical analyses were performed with GraphPad Prism (version 8). A paired-sample t-test was employed to evaluate the significance of the data for each concentration of the tested drug platforms with biofilm formation levels. The analysis of variance (ANOVA) was used to determine whether the groups under investigation differed significantly. *P*-values below 0.05 were considered statistically significant.

## Results

### Synthesis and characterization of GNSO, GSNO-PVMMA, and copolymer

**Fig. 1:**
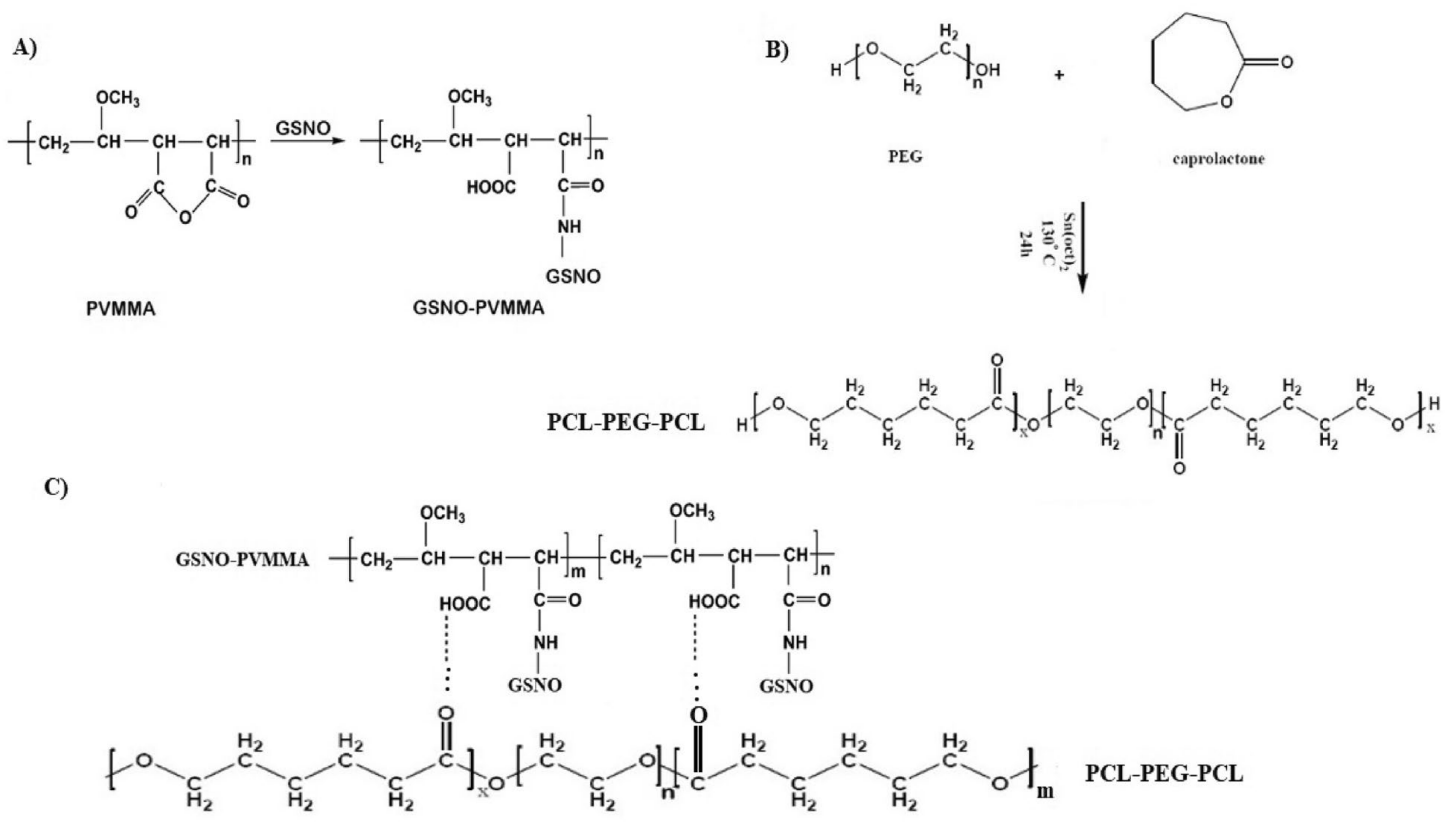
Scheme of the synthesis of (**A**) PVMMA-GNSO, (**B**) PCL–PEG–PCL, and (**C**) hydrogel

**Fig. 2:**
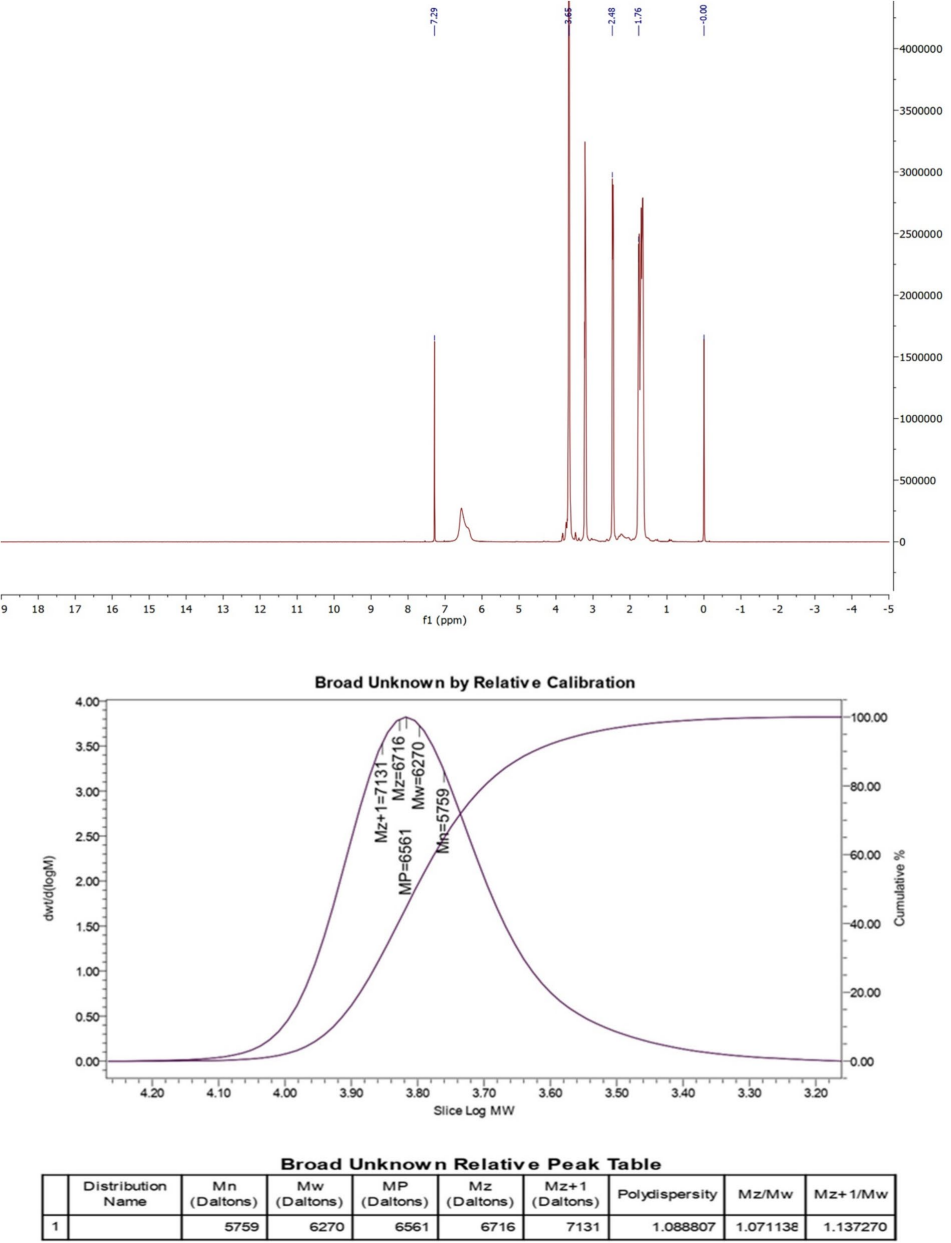
Characterization of GNSO, GSNO-PVMMA, and copolymer. **A** H-NMR of PCL–PEG–PCL copolymer, (**B**) GPC of PCL–PEG–PCL copolymer

**Fig. 3:**
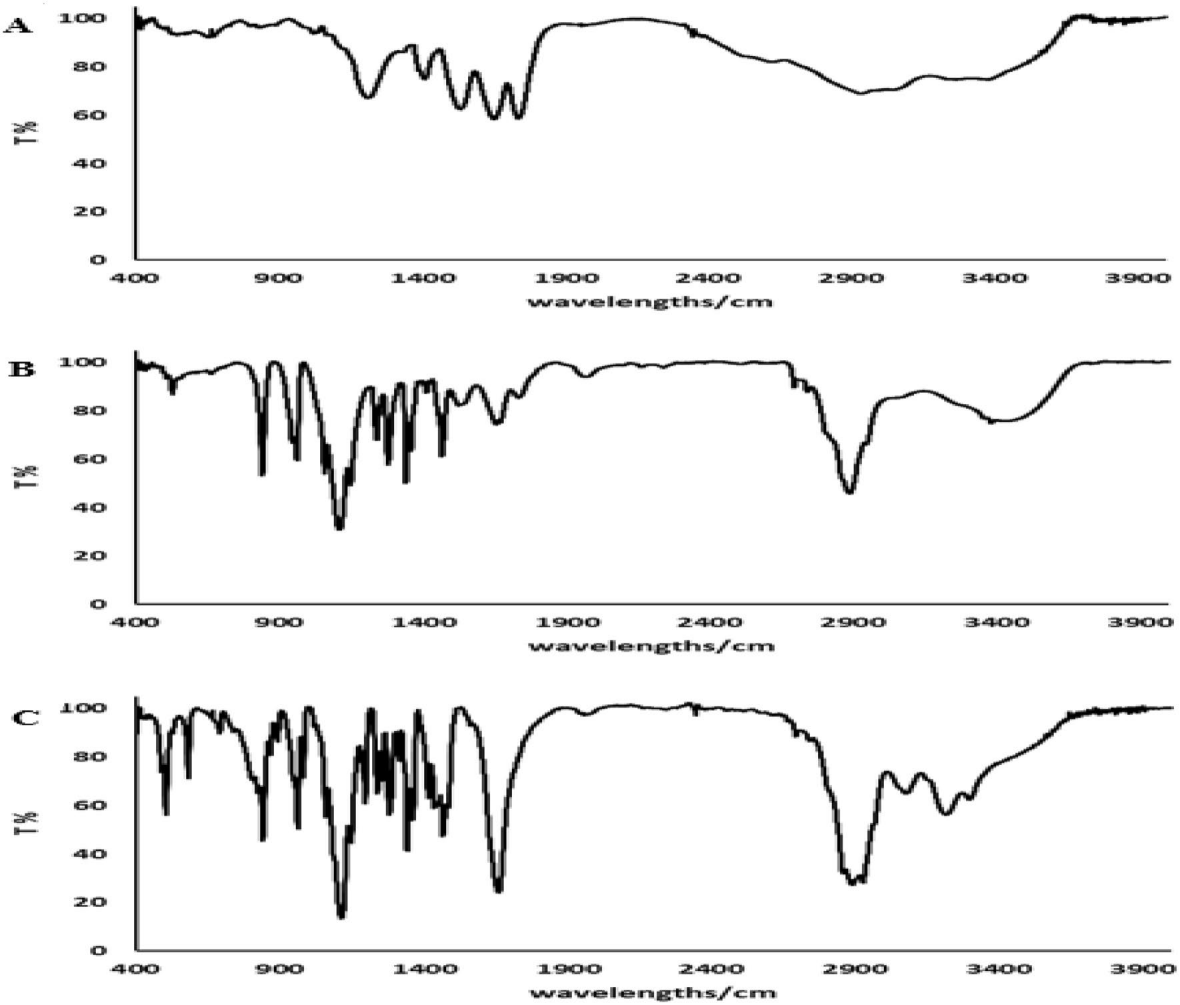
FTIR spectroscopy of (**A**) GSNO, (**B**) PVMMA-GSNO, and (**C**) PCL–PEG–PCL

We produced a PCL–PEG–PCL copolymer utilizing the specified approach [17]. The scheme of PCL–PEG–PCL synthesis is shown in Fig. 1. The molecular structure of PCL–PEG–PCL was examined by H-NMR utilizing CDCl3 as the solvent (Fig. 2). The distinctive peaks of the PCL–PEG–PCL moiety were detected at *δ* = 3.65 ppm (–OCH_2_–CH_2_–O) and *δ* = 1.83 ppm [C–(CH_3_)]. The chemical shifts of the PCL segment were observed at *δ* = 2.6 ppm [O = C–O–(CH2)] and *δ* = 3.2 ppm (CH_2_–CH_2_). The synthesized PCL–PEG–PCL, GNSO, and GSNO-PVMMA were analyzed by FTIR (Fig. 3). A signal at 1214 cm−1 was associated with the C–O–C functional groups. Furthermore, the prominent peaks at 2900 and 1410 cm−1 correspond to the C–H stretching and bending modes, respectively. The absorption band about 1680 cm−1 was associated with the stretching vibration of carbonyl (C = O) groups. The molecular weights (Mn and Mw) and polydispersity index (PDI) of the copolymer, determined using GPC, were 5759 Daltons, 6270 Daltons, and 1.089, respectively (Fig. 2). The estimated molecular weight determined by H-NMR spectroscopy was around 5947 Daltons.

### Hydrogels characteristics

**Fig. 4:**
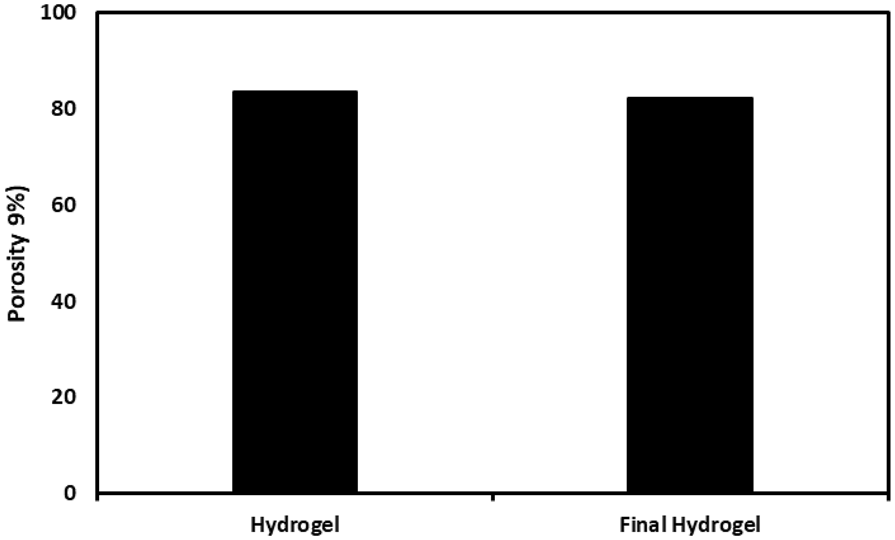
Porosity percentage of the pure hydrogel and drug/NP-loaded hydrogel. The porosity of the hydrogels is in the range of 80–90%

The results showed that the fabricated hydrogels have a porous structure and interconnected pores. The EDX analysis of the hydrogel complex verified the existence of AgNPs within the hydrogel. Furthermore, this analysis demonstrated that the hydrogel exhibited high purity and lacked additional chemical contaminants. SEM was used to examine the hydrogel’s surface morphological properties and its AgNPs dispersion. According to the analysis, the hydrogel’s and the final hydrogel’s surfaces were clear and cracked (Supplementary File, Figure S1). Further, although the final hydrogel had a high aggregation of AgNPs, the results indicated that the AgNPs were evenly distributed throughout the hydrogel network. The elemental peaks of C, O, and Ag were detected in the EDS spectra at around 0.2 keV, 0.5 keV, and 3.0 keV, respectively. The C, S, and O peaks represent the hydrogel matrix, whereas the Ag peak confirms the presence of nanocomposites. The presence of a porous network structure is essential for the process of drug loading and subsequent release of bioactive substances (Fig. 4). The results showed that the porosity of the hydrogels is in the range of 80–90%. The mechanical property test findings indicated that adding AgNPs and drugs reduces the tensile strength of the pure hydrogel, causing it to decrease from 4.45 to 0.73 MPa.

### Swelling/deswelling cycle

**Fig. 5:**
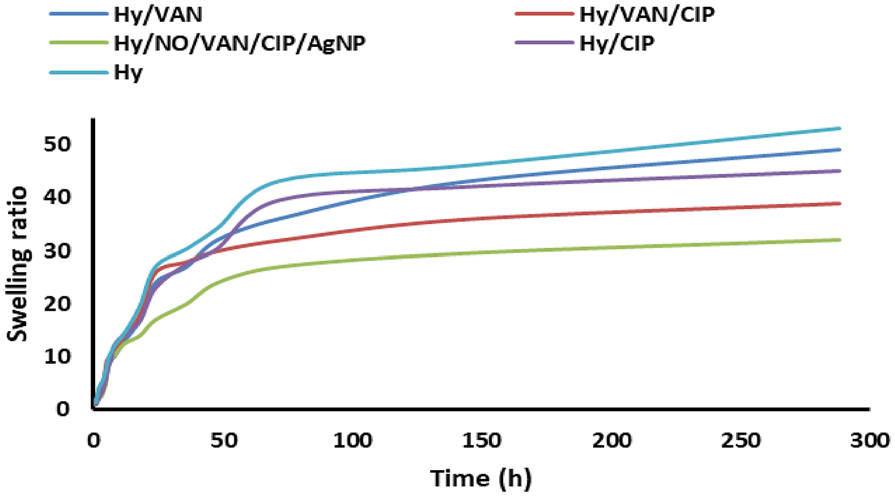
The swelling data of the synthetized hydrogel

The swelling was conducted to assess the reversibility of the hydrogel’s swelling behavior (Fig. 5). Incorporating the drugs and nanoparticles into the hydrogel reduced the swelling ratio compared to the standard hydrogel. The observed kinetic data indicated that the integration of the mentioned compounds adversely affected the expansion of the hydrogel network due to their rigidity and substantial volume, which diminished the network’s flexibility. We determined that the controlled thermoresponsive characteristic was preserved throughout the swelling cycle, indicating the structural stability of the hydrogel, which is essential for future practical applications [31].

### Drug loading and release kinetics

The hydrogel loading was steady and exhibited a continual increase. Drugs and AgNPs were included in the hydrogel, enhancing drug solubility. Consequently, an increased drug loading was achieved compared to the standard hydrogel (Table 2). This outcome demonstrated that the medication loading was effective.

**Table 2:** The amount of drugs and AgNPs loaded in the formulation

Sample	Hydrogel/vancomycin	Hydrogel/ciprofloxacin	Hydrogel/AgNPs	HyNSVC
Vancomycin	32 mg/ml	–	–	32 mg/ml
Ciprofloxacin	–	8 mg/ml	–	8 mg/ml
AgNPs	–	–	29.8 ppm	29.8 ppm

**Fig. 6:**
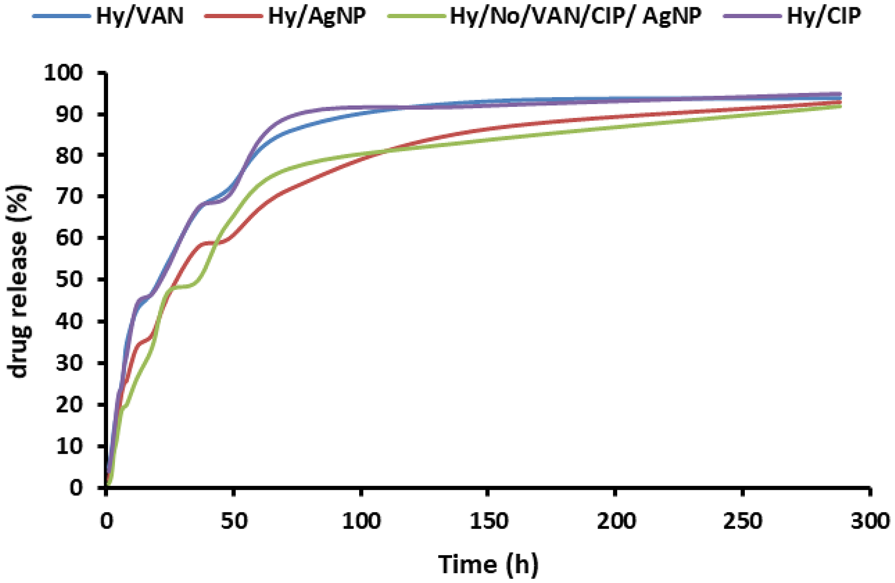
In vitro release profiles of vancomycin, ciprofloxacin, and AgNPs from hydrogels

As illustrated in Fig. 6, the liberation of drugs and AgNPs from hydrogels escalated with time. This finding suggests that the release of the mentioned compounds from hydrogels can be controlled by time and circumstances. The data indicate that the release of drugs and AgNPs from the hydrogel was contingent upon the presence of drug molecules and environmental conditions. The presence of different agents and nanoparticles in the hydrogel influenced matrix swelling, drug dissolution, and diffusion, which might occur concurrently.

### Toxicity assay

**Fig. 7:**
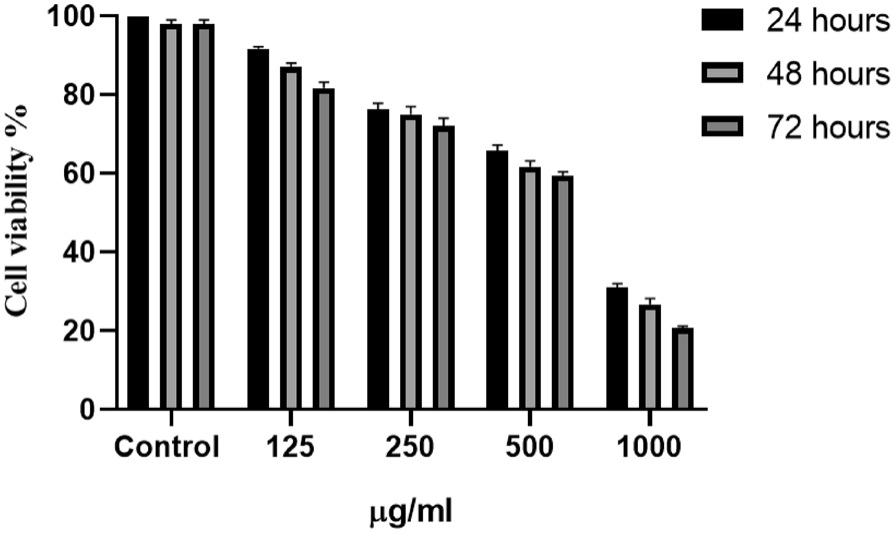
In vitro cytotoxicity effect of synthesized HyNSVC on the A549 cell line. Results indicated that at the MIC concentration of HyNSVC, 78.75% of the cells remained alive

The cell viability of HyNSVC at various doses was assessed on the A549 cell line (Fig. 7). The MTT test results indicated that at the MIC concentration of HyNSVC, 78.75% of the cells remained alive.

### Bacterial isolation and biofilm formation ability

The antibiogram findings indicated that the isolated *P. aeruginosa* was susceptible to ciprofloxacin and carbapenems. In addition, the MTP method showed five and two isolated MRSA and *P. aeruginosa* produced strong biofilms compared to the standard strains, respectively (Supplementary File, Figure S2, Tables S1 and S2).

### The antibacterial and antibiofilm activity of HyNSVC

In this study, the MIC of HyNSVC was 250 μg/ml (8 μg/ml vancomycin, 2 μg/ml ciprofloxacin, 7.4 μg/ml AgNPs) against MRSA and *P. aeruginosa* strains. The HyNSVC showed the lowest MIC against both bacteria compared to other drug platforms. On the other hand, free Hy and Hy–NO did not show antibacterial effects against bacteria (Table 3).

**Table 3:** The MIC concentration of different drug platforms

Isolate	Hy and Hy-NO	HY-CIP	HY-Van	HY-AgNPs	HY-NO-AgNPs	HY-NO-AgNPs-VAN	HY-NO-AgNPs-CIP	HY-NO-VAN-CIP	HyNSVC
*MRSA*	–	1000 µg/mL	1000 µg/mL	1000 µg/mL	1000 µg/mL	500 µg/mL	500 µg/mL	500 µg/mL	250 µg/mL
*P. aeruginosa*	–	500 µg/mL	–	1000 µg/mL	1000 µg/mL	1000 µg/mL	250 µg/mL	500 µg/mL	250 µg/mL

**Fig. 8:**
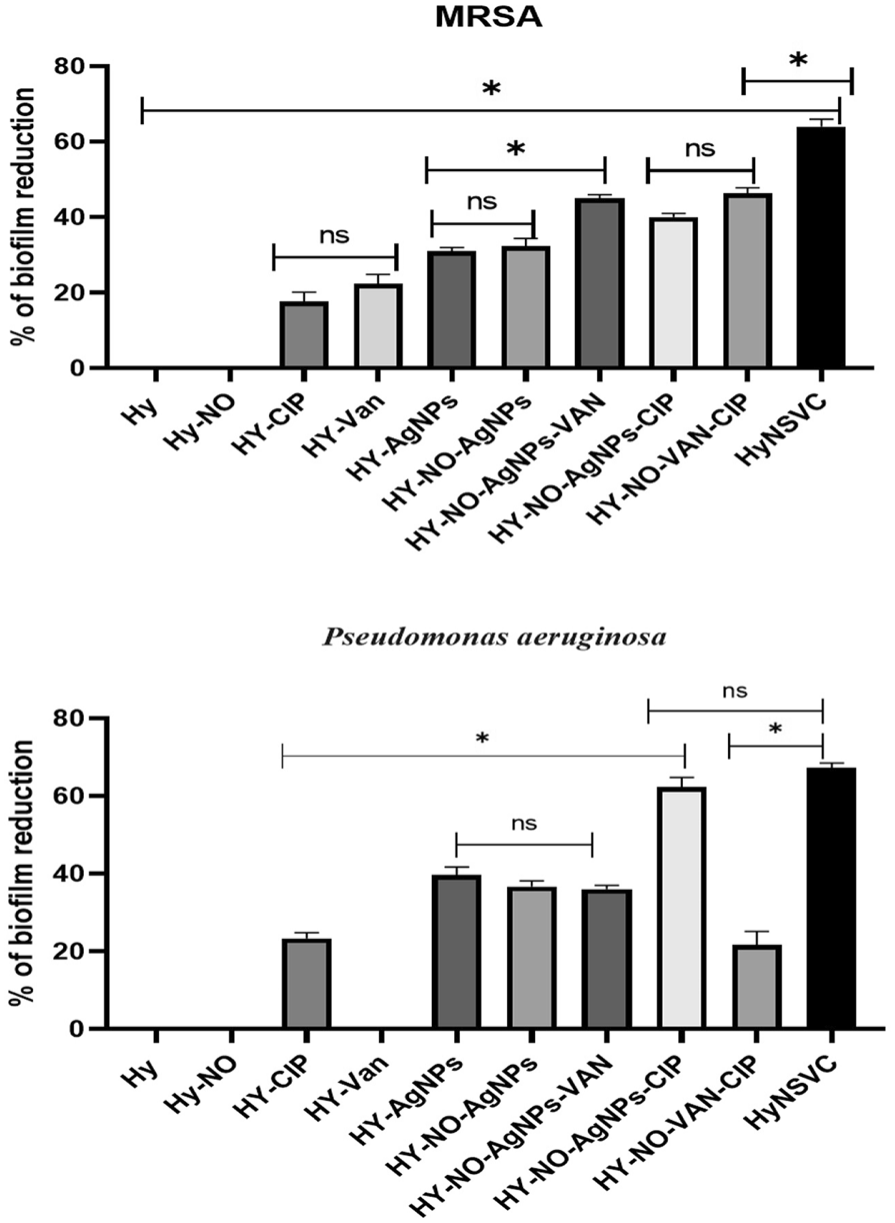
The percentage of mature biofilm reduction after treatment with 2 × MIC of drug platforms (mean ± SD, *n* = 3, ns: insignificant, *p < 0.05)

**Fig. 9:**
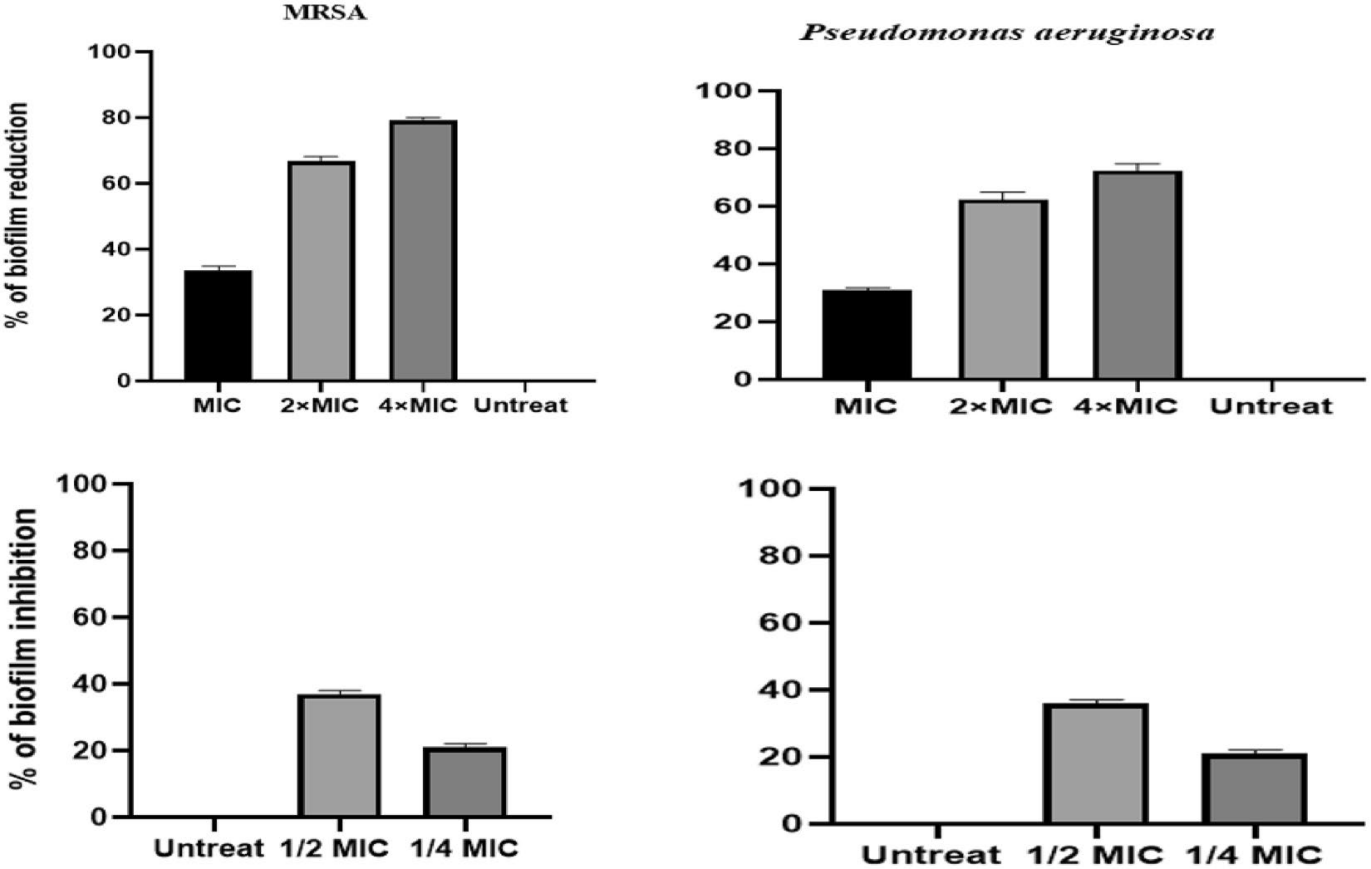
Antibiofilm effect of different concentrations of HyNSVC against MRSA and *P. aeruginosa*

The MTP experiment demonstrated that the HyNSVC formulation significantly destroyed mature biofilm in both bacteria compared to other drug platforms (Fig. 8). The findings indicated that sub-MIC of HyNSVC (125 µg/ml) more effectively inhibited biofilm formation and reduced OD values in both bacterial strains compared to other drug platforms (*P* < 0.05). In addition, the results indicated that the 2 × MIC of HyNSVC resulted in a substantial decrease in mature biofilm, with reductions of 62% in *P. aeruginosa* and 68.1% in MRSA detachment (Fig. 9).

### The effect of HyNSVC on the expression of biofilm-associated genes

**Fig. 10:**
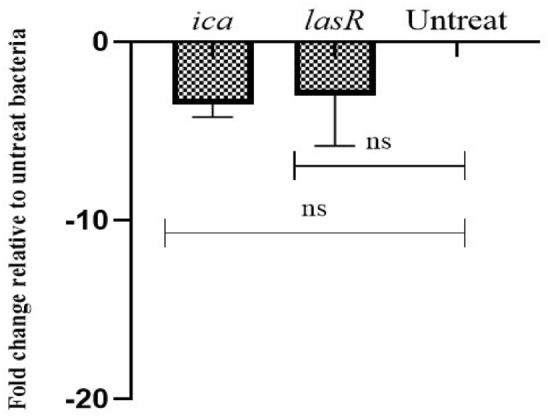
The fold change relative to untreated clinical isolates of *P. aeruginosa* and MRSA (mean ± SD, *n* = 3, ns: insignificant)

The findings indicated a higher expression ratio of biofilm-associated genes in clinical isolates relative to standard strains (Fig. 10). The expression levels of the *icaA* gene for MRSA and the *lasR* and *algD* genes for *P. aeruginosa* were quantified in the presence of HyNSVC. The HyNSVC did not significantly diminish the expression of all genes compared to the untreated bacteria; *p* > 0.05.

## Discussion

Persistent CRS presents a significant therapeutic challenge in otolaryngology owing to its multifactorial etiology. Factors including polyposis, mucosal edema, and secretions are physical impediments that influence the drug’s efficacy, retention, and administration to the targeted sinus. Even when drugs arrive at the site of inflammation, ciliary clearance rapidly removes them from the mucosal surface, hindering sufficient mucosal contact time with the drug [32]. In addition to its inadequate concentration for eliminating bacteria in the sinonasal biofilm, systemic antibiotic therapy frequently induces antibiotic resistance and disrupts the natural microbiota in patients. Consequently, developing alternate therapeutic strategies to systemic antibiotic therapy is underscored in managing bacterial biofilm in patients with CRS [8].

As mentioned, the main aim of this study was to use hydrogels for local treatment due to their high mucosal binding ability and delivery of antibacterial and antibiofilm agents; therefore, the properties of the hydrogel matrix were very important. The FESEM and EDX data for HyNSVC display the hydrogel’s porous architecture and elemental distribution, respectively. These analyses confirm both the morphological characteristics and the successful incorporation of Ag, N, and O elements consistent with the composite formulation. The results showed that the porosity of the hydrogels is in the range of 80–90%. The mechanical property test findings indicated that adding AgNPs and drugs reduces the tensile strength of the pure hydrogel. The hydrogel loading was steady and exhibited a continual increase. Also, the finding suggests that the release of the mentioned compounds from hydrogels can be controlled by time and circumstances. Results indicated that at the MIC concentration of HyNSVC, 78.75% of the cells remained alive. According to all the analyses, we successfully fabricated a porous hydrogel matrix from PCL and PEG in the presence of AgNPs and drugs, considering potential applications of drug delivery in sinuses.

Combining the compounds in the hydrogel has led to increased effectiveness of AgNPs and antibiotics compared to their individual use. Generally, another goal of the present study was to design a structure that could deliver effective antibiotics such as vancomycin, which cannot be used locally and in systemic form; the required concentration may not reach the sinus area. Also, ciprofloxacin is a frequently recommended antibiotic for CRS; nevertheless, the capacity for targeted release in the sinuses may reduce adverse effects and enhance medication concentrations at the diseased location. Therapeutic strategies that provide high concentrations of antibiotics at affected locations for prolonged durations might effectively eliminate bacteria encased in biofilms while mitigating the unfavorable effects of prolonged systemic antibiotic therapy [33]. The hydrogel can be a means of delivering drugs to the sinus area for maintaining and releasing them for a long time.

Our findings revealed that the HyNSVC showed the MIC at 250 μg/ml on MRSA and *P. aeruginosa* strains. On the other hand, free Hy and Hy-No did not show antibacterial effects against MRSA and *P. aeruginosa* strains. Compared to the Hy-Ag, Hy-vancomycin, and Hy-ciprofloxacin, a 2- to 4 fold decrease in MIC was observed in the HyNSVC. Also, the results indicated that the 2 × MIC of HyNSVC resulted in a substantial reduction in mature biofilm, with a decrease of 62% in *P. aeruginosa* and 68.1% in MRSA.

In line with our findings, the study by *Yathavan* et al. also showed that hydrogels containing AgNPs capable of releasing sustained silver ions for three days had potent antibacterial activity in vitro against *P. aeruginosa* and *S. aureus*. The results indicated that in situ generating thermoresponsive hydrogel networks present a viable approach for the localized and regulated administration of AgNPs within the sinonasal cavity [34]. In another study, a self-healing polyethylene glycol hydrogel therapeutic model was evaluated to promote locally sustained drug release and prevent adverse effects. The findings indicated that polyethylene glycol hydrogel preserves self-healing properties, biodegradability, a moderate swelling rate, injectability, and prolonged drug release [35]. Also, in another article in 2020, chitosan/polyvinylpyrrolidone hydrogel scaffolds containing PLGA microparticles loaded with dexamethasone were used to inhibit CRS bacteria (*S. aureus* and *E. coli*). Their findings indicated that hydrogels exhibit antibacterial efficacy. Notably, compared to using nanoparticles and the drug alone, the hydrogel showed slower and longer release ability [36]. Moreover, *Jalessi* et al. assessed the prolonged co-release of encapsulated ciprofloxacin and dexamethasone (DEX) in polyvinyl alcohol-based carriers within the maxillary sinus of rabbits. This drug delivery method was observed to facilitate effective, regulated, and safe sustained drug administration in both in vitro and in vivo studies at therapeutic concentrations, with limited systemic absorption, indicating a viable therapy strategy for CRS. The researchers highlighted that their platform requires assessment in an animal model of CRS with bacterial infections, specifically *S. aureus* and *P. aeruginosa*, which are prevalent pathogens in CRS—the impact of synthesized drug platforms on biofilms warrants examination in a distinct investigation [32].

The authors proposed that slow and sustained release from hydrogel could be a long-term antibiotic delivery and increase antibiotic antibacterial activity and penetration to the deeper layers of biofilm. A notable point in our study is the presence of AgNPs, vancomycin, and ciprofloxacin and their interaction. The antibacterial and antibiofilm efficacy of AgNPs against a wide range of microorganisms has been progressively utilized in multiple domains [37]. However, AgNPs are readily oxidized and/or aggregated in air, which impedes their practical utilization [38]. For this reason, in the previous study, we used the green product phycocyanin as a protective agent for synthesizing AgNPs (Ag-Pc). Then, this Ag-Pc was loaded in HyNSVC. Nonetheless, the mechanisms through which AgNPs exert their antibacterial effects remain inadequately understood. It is posited that biologically active Ag⁺ ions are released in an aqueous solution, hence enhancing the antibacterial impact [39]. AgNPs engage with cellular components such as cell walls, the cytoplasmic membrane, and ribosomal DNA [40]. The possible interaction mechanism between vancomycin and AgNPs is related to the cell wall. The results of a study indicated that vancomycin shows a synergetic antibacterial effect against both Gram-positive and Gram-negative strains in combination with AgNPs. The authors have assumed that a reaction occurs between vancomycin and the surface of the AgNPs. The formed complex binds to the bacteria; at this time, the silver ions released from the complex bind to the bacterial cell wall [39].

In addition, we believe that in our study, the penetration and effectiveness of ciprofloxacin will increase after the interaction between AgNPs and vancomycin. Ciprofloxacin binds to and inhibits DNA gyrase enzymes, modulating bacterial DNA supercoiling. However, ciprofloxacin resistance is growing among certain bacteria [41]. Resistance may arise from the activity of efflux pumps or mutations in DNA gyrase genes. The specific interaction between metal nanoparticles and ciprofloxacin has not yet been documented. However, it seems that when these nanoparticles attach to the bacterial cell membrane, they create holes in the bacterial cell walls and harm the cell membrane, which makes it easier for ciprofloxacin to enter the bacterial cells’ periplasm [42].

Our results also showed the inhibitory effect of the synthesized hydrogel against bacterial biofilm. Despite several studies examining the inhibitory effects of AgNPs on bacterial biofilms, the interactions between these biofilms and AgNPs remain inadequately elucidated. Despite the intricacy of bacterial biofilms, certain overarching insights may be articulated regarding the primary physicochemical variables that govern the NP–biofilm interaction. The principal interactions are hydrophobic, steric, and electrostatic [43]. The interactions mostly involve physical processes; however, chemical and biological interactions may also occur between the EPS matrix and NPs. Electrostatic interactions are crucial in regulating biofilm formation, particularly during the initial adherence to surfaces and the subsequent cohesiveness of the EPS matrix [44]. Moreover, hydrophobic interactions significantly influence biofilm development and its subsequent management [45]. Steric interactions are crucial, especially for the colloidal stability of NPs; steric stabilization can inhibit nanoparticle aggregation, significantly influencing interactions with biofilm extracellular polymeric substances [46]. Generally, positively charged NPs exhibit a greater propensity to interact with EPS, such as polysaccharide matrices, proteins, and DNA, which predominantly possess a negative charge [47]. A prevalent example is the use of AgNPs, which frequently exhibit enhanced inhibitory effects on microbial colonization, adhesion, and biofilm formation. Despite several studies examining the inhibitory effects of AgNPs on bacterial biofilms, the interactions between these biofilms and AgNPs remain inadequately elucidated [43]. However, considering the interaction between AgNPs and biofilm, it is likely that the access and entry of both antibiotics, vancomycin and ciprofloxacin, into the biofilm community and its internal structure will increase, which in turn will be effective in combating and eradicating the microbial population within the biofilm.

We could not observe the Hy-NO group's desired effect on biofilm in vitro. We believe that examining the effect of this platform in vivo and the reduction of NO in the mucus of individuals with CRS will better demonstrate the expected results regarding the effect on the biofilm community. Therefore, the impact of this Hy-NO can be investigated in future studies on a suitable animal model. In the end, our findings suggested that bacterial biofilm treatment with sub-MIC doses of HyNSVC had no significant impact on the expression of biofilm-associated genes. Consequently, additional confirmatory investigations are required to enhance understanding of the connection between HyNSVC and the gene expression related to bacterial adhesion and biofilm community formation.

## Conclusion

This study found that hydrogels enhanced the pharmaceutical index of free drugs and AgNPs, suggesting they could deliver antimicrobial medications to the sinus cavity. Furthermore, the HyNSVC system enhanced the antibacterial activity of free drugs and AgNPs, which might be developed into a drug delivery system that effectively combats clinical isolates of *P. aeruginosa* and MRSA with minimal cytotoxic effects. This formulation integrates antibiotic and anti-inflammatory agents into one product, rendering it particularly useful for managing CRS while allowing for the administration of traditional antibiotics at reduced yet effective dosages, minimizing unwanted effects.

However, further studies, especially in vivo evaluation, are necessary to evaluate the drug delivery capabilities and interaction of the hydrogel and its components with biofilm. These evaluations could provide valuable insights for the clinical development of this drug delivery system in combating bacterial infections, especially in patients with CRS. Also, there was not enough time in this study to collect more clinical isolates. Future studies will assess antibiotic resistance and biofilm formation in more isolates over a longer period of time.

## Supplementary Information

Supplementary Material 1

## Data Availability

The authors confirm that the data supporting the findings of this study are available within the article.
