# Supplementary material for: The effectiveness of hydrogel matrix containing nitric oxide, silver nanoparticles, vancomycin, and ciprofloxacin on methicillin-resistant Staphylococcus aureus and Pseudomonas aeruginosa biofilm isolated from patients with chronic rhinosinusitis
Source: Eur J Med Res. 2025 Oct 29;30:1036. doi: 10.1186/s40001-025-03282-z (PMC12570856; doi:10.1186/s40001-025-03282-z)
Supplement: Supplementary file 1 — Supplementary Material 1 [file 40001_2025_3282_MOESM1_ESM.docx]

A)
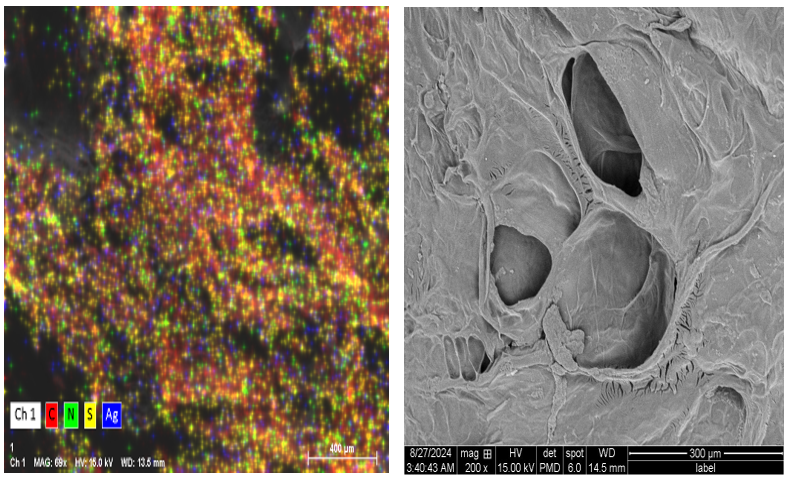


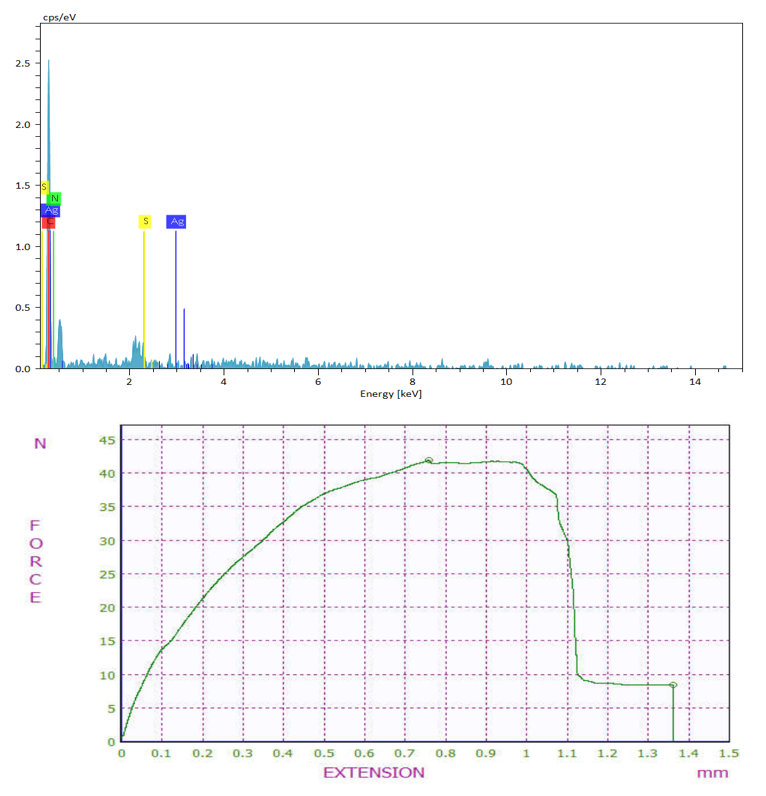


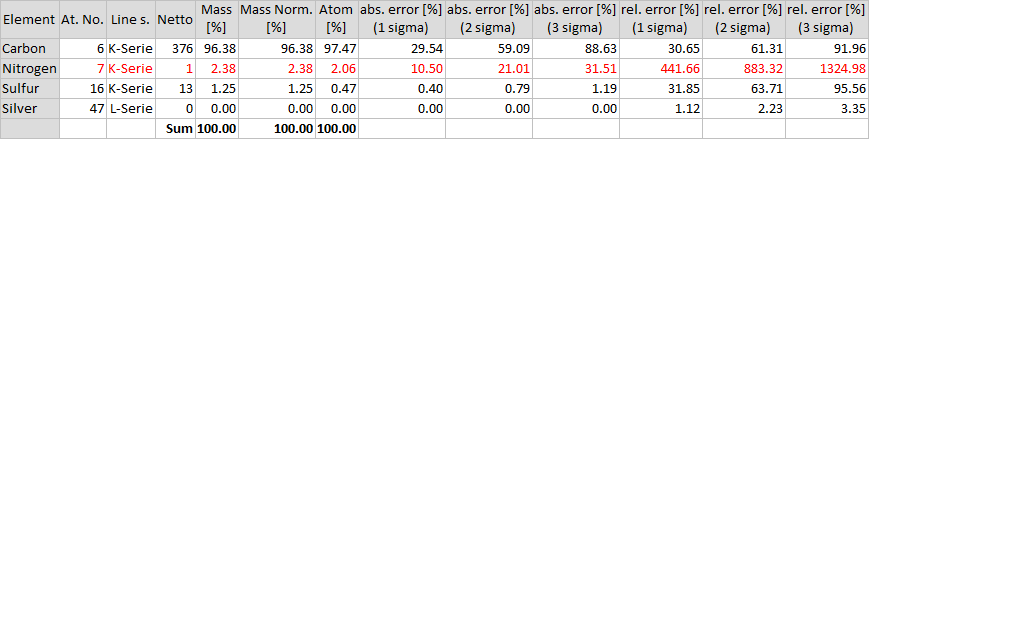


B)


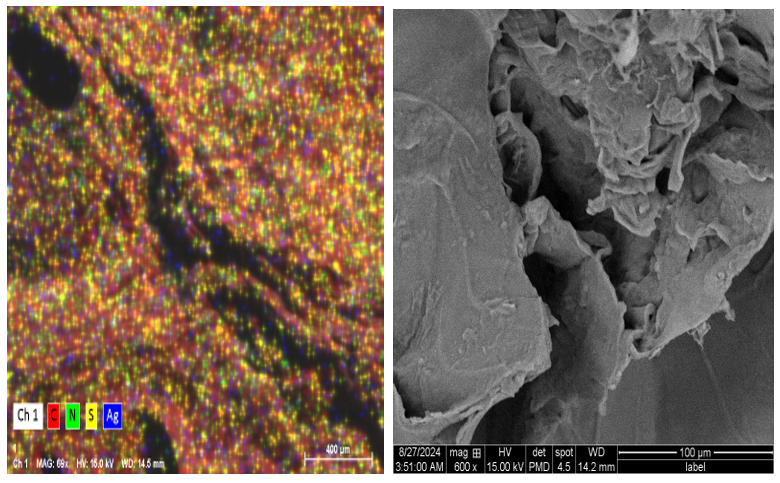


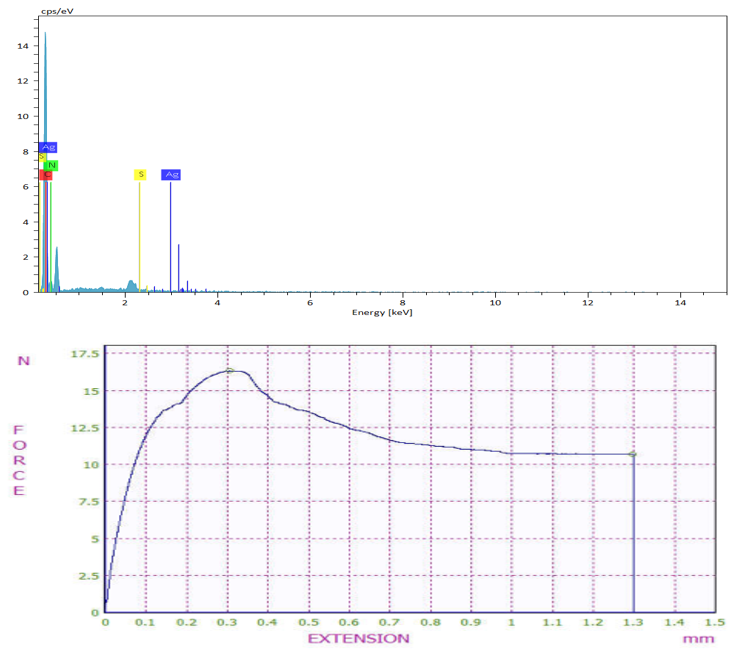


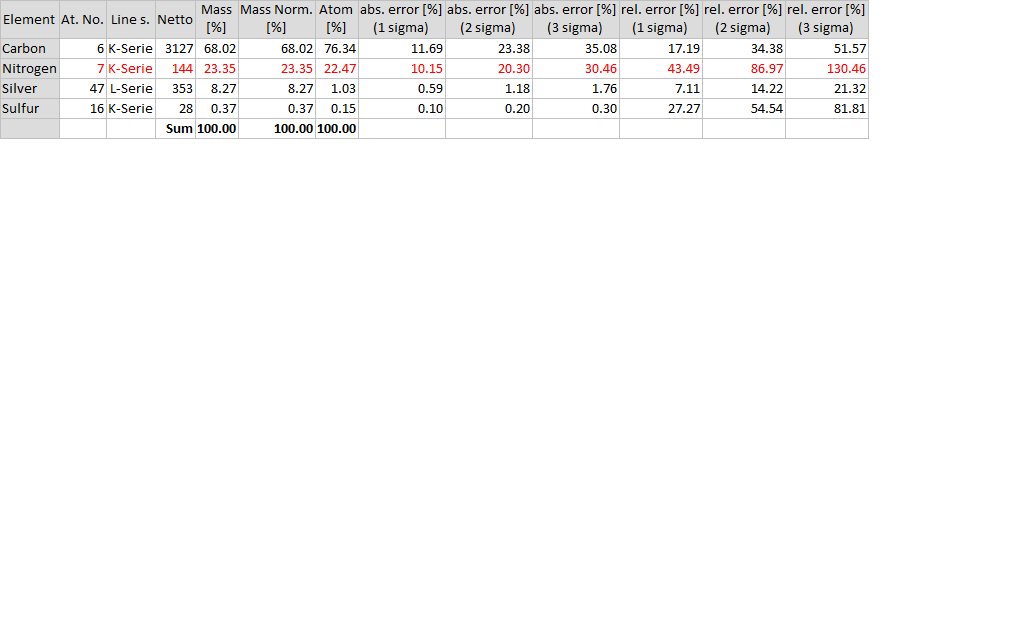


C)


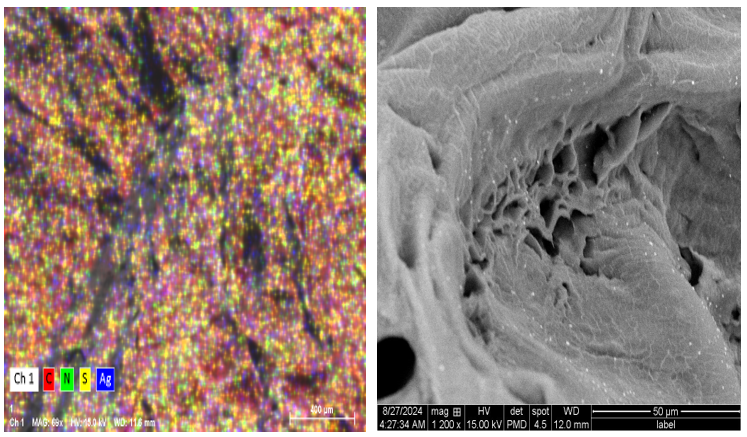


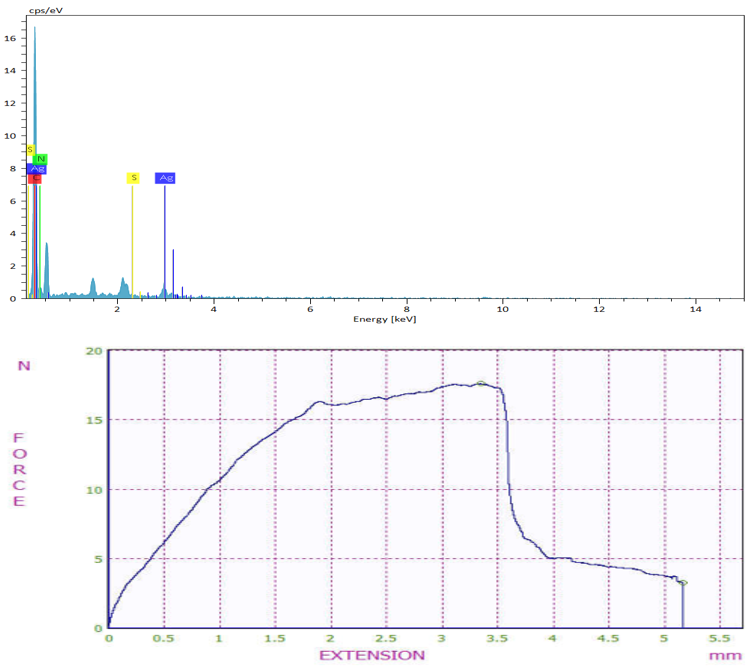


**Figure S1.** (A) SEM micrograph, EDS, and tensile of the fabricated pure hydrogel (B) drug-loaded hydrogel and AgNPs (C) drug-loaded hydrogel. Scale bar: 300 µm.

Table S1. Characteristics of strains isolated from patients.

| Isolate  (n=10) | Bacteria | Biofilm formation |
| --- | --- | --- |
| A1 | MSSA | Strong |
| A2 | VRSA | Strong |
| A3 | MRSA | Strong |
| A4 | MSSA | Moderate |
| A5 | MRSA | Strong |
| A6 | MSSA | Weak -biofilm forming |
| A7 | MRSA | Strong |
| P1 | Cipro-S | Strong |
| P2 | Cipro-S | Weak -biofilm forming |
| P3 | Cipro-S | Strong |

MRSA: methicillin-resistant *Staphylococcus aureus*; MSSA: methicillin-sensitive *Staphylococcus aureus*. VRSA: vancomycin-resistant *Staphylococcus aureus;* Cipro-S: Ciprofloxacin-sensitive

Table S2. Antibiogram results of isolates.

| Strains (n = 10) | *P. aeruginosa*  (n=3) | MRSA  (n=3) | MSSA  (n=3) |
| --- | --- | --- | --- |
| Ciprofloxacin | S | - | - |
| Levofloxacin | I | - | - |
| Imipenem | S | - | - |
| Ceftazidime | S | - | - |
| Piperacillin | S | - | - |
| Tobramycin | S | - | - |
| Cefoxitin | - | R | S |
| Gentamycin | - | S | S |
| Clindamycin | - | R | S |
| Mupirocin | - | S | S |
| Amoxicillin | - | R | S |
| Trimethoprim/sulfamethoxazole | - | R | S |
| Erythromycin | - | R | R |

MRSA: methicillin-resistant *Staphylococcus aureus*; MSSA: methicillin-sensitive *Staphylococcus aureus*. VRSA: vancomycin-resistant *Staphylococcus aureus*


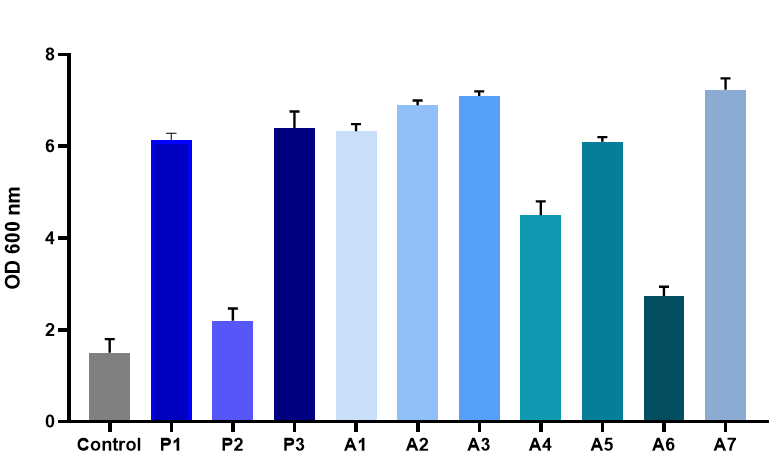


**Figure S2.** the biofilm production of bacterial isolates.

Table S3.  16S rDNA gene-targeted specific primers and TaqMan probes for isolation of bacteria.

| Target Bacteria | Primer/probe* | Oligonucleotide sequence |
| --- | --- | --- |
| *Staphylococcus aureus* | Primer F  Primer R  Probe | AAGTCGAGCGAACGGACGAG  CTCTCAGGTCGGCTATGCATCG  ACCTTACCAACTAGCTAATGCAGCGCGGAT |
| *Pseudomonas aeruginosa* | Primer F  Primer R  Probe | GTGGTTCAGCAAGTTGGATGTG  CCACGCTTTCGCACCTCAG  CGCCTTCGCCACTGGTGTTCCTTCCTATA |

*Primers F (forward), R (reverse) and probes targeting the 16S rDNA gene

**
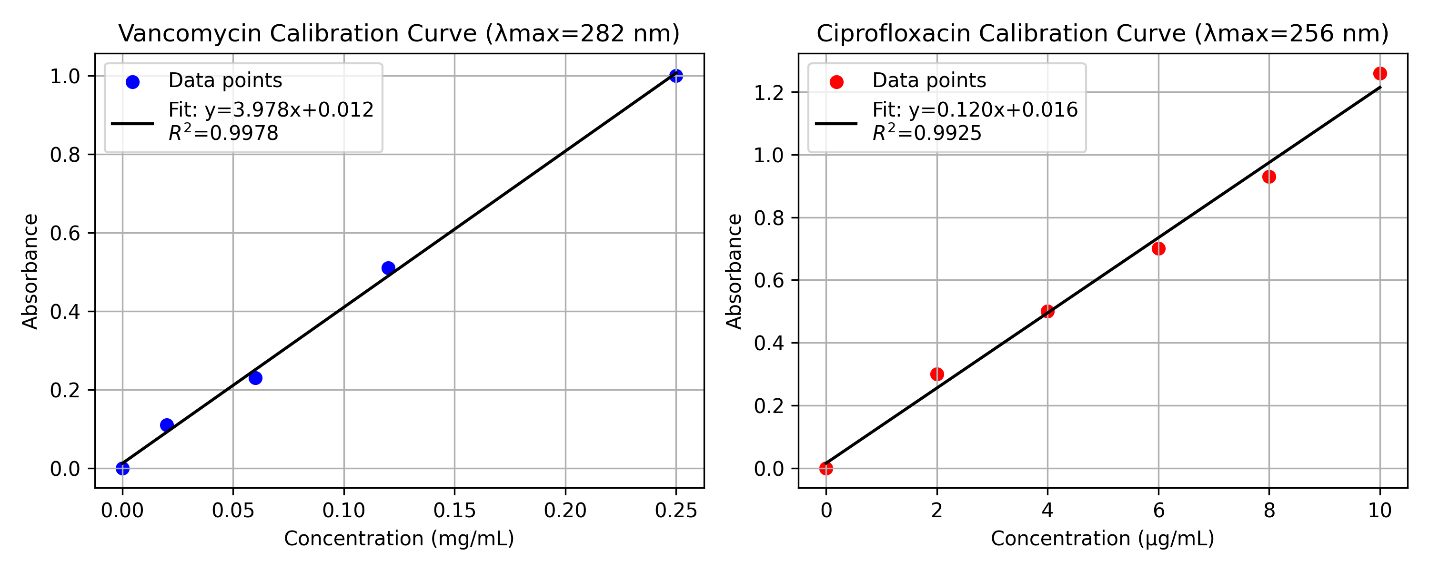
**

**Figure S3**. Standard carve of Ciprofloxacin and Vancomycin.
